# A cross-sectional study identifying the pattern of factors related to psychological intimate partner violence exposure in Slovenian family practice attendees: what hurt them the most

**DOI:** 10.1186/1471-2458-14-223

**Published:** 2014-03-04

**Authors:** Polona Selic, Igor Svab, Nena Kopcavar Gucek

**Affiliations:** 1 Department of Family Medicine, Faculty of Medicine, University of Ljubljana, Poljanski nasip 58, Ljubljana, Slovenia

**Keywords:** Intimate partner violence, Psychological abuse, Employment status, Disputes in intimate relationship, Level of education

## Abstract

**Background:**

Intimate partner violence (IPV) is yet to be fully acknowledged as a public health problem in Slovenia. This study aimed to explore the health and other patient characteristics associated with psychological IPV exposure and gender-related specificity in family clinic attendees.

**Methods:**

In a multi-centre cross-sectional study, 960 family practice attendees aged 18 years and above were recruited. In 689 interviews with currently- or previously-partnered patients, the short form of A Domestic Violence Exposure Questionnaire and additional questions about behavioural patterns of exposure to psychological abuse in the past year were given. General practitioners (GPs) reviewed the medical charts of 470 patients who met the IPV exposure criteria. The Domestic Violence Exposure Medical Chart Check List was used, collecting data on the patients’ lives and physical, sexual and reproductive, and psychological health status, as well as sick leave, hospitalisation, visits to family practices and referrals to other clinical specialists in the past year. In multivariate logistic regression modelling the factors associated with past year psychological IPV exposure were identified, with *P* < 0.05 set as the level of statistical significance.

**Results:**

Of the participants (n = 470), 12.1% (n = 57) were exposed to psychological IPV in the previous year (46 women and 11 men). They expressed more complaints regarding sexual and reproductive (p = 0.011), and psychological and behavioural status (p <0.001), in the year prior to the survey. Unemployment or working part-time, a college degree, an intimate relationship of six years or more and a history of disputes in the intimate relationship, increased the odds of psychological IPV exposure in the sample, explaining 41% of the variance. In females, unemployment and a history of disputes in the intimate relationship explained 43% of the variance.

**Conclusions:**

The prevalence of psychological IPV above 10% during the past year was similar to earlier studies in Slovenia, although the predominance of better-educated people might be associated with lower tolerance toward psychological abuse. GPs should pay special attention to unemployed patients and those complaining about family disputes, to increase early detection.

## Background

There has been increasing recognition in Slovenia of the societal implications of intimate partner violence (IPV), defined as “behaviour within an intimate relationship that causes physical, sexual or psychological harm, including acts of physical aggression, sexual coercion, psychological abuse and controlling behaviours” [1]. The reported prevalence in Slovenia [2-4], as well as a growing body of research on health consequences related to IPV [5-11] and its impact on morbidity and mortality [5], all suggest a gradual change from IPV being seen as a personal and family issue related to the legal and justice system, to an issue that needs to be acknowledged and addressed as a public health problem.

Women have traditionally been considered the victims and men the perpetrators of IPV [12,13]; this is probably due to findings that men use more physical force and sexual coercion and are more likely to cause injuries [14-18]. However, women were reported to be more likely to employ psychological aggression [19]. In a limited body of research, it has been suggested that female perpetrators are more likely to suffer from mental health difficulties, especially anxiety and depression [20,21].

Although knowledge of the comparative health effects of different types of IPV is limited, some studies have indicated that victims’ exposure to psychological aggression may be more strongly associated with the onset of depression, anxiety, somatisation, and post-traumatic stress disorder than other types of IPV [9,14,20,22-24]. Coker et al. [14] reported both physical and psychological IPV to be associated with significant physical and mental health consequences in victims regardless of gender. Women were significantly more likely than men to experience and report physical or sexual IPV and abuse of power and control, but less likely than men to report verbal abuse alone. For both men and women, being a victim of physical IPV was associated with increased risk of current poor health. In general, abuse of power and control was found to be more strongly associated with adverse health consequences than verbal abuse, and psychological violence was shown to be more damaging to overall health status than physical abuse [14].

In another study, Coker et al. [25] explored the consequences of IPV on physical health and found that women experiencing psychological IPV were significantly more likely to report poor physical and mental health. Psychological IPV was as strongly associated with the majority of adverse health outcomes as physical IPV [25]. Summarising the findings of existing studies, the adverse effects of IPV on women’s health should be noted, such as chronic pain [5,14,26], chronic stress-related symptoms [27] and central nervous system problems [5,14]. Authors worldwide mostly agree in their findings on both the short and long term physical and mental health consequences of IPV exposure [5,28,29]. IPV exposure has been found to be associated with an increase in psychoactive substance use, anxiety, depression, suicidality, and post-traumatic stress disorder symptoms [14,30-36].

Undoubtedly both men’s and women’s IPV exposure should be explored in IPV research, but the findings need to be interpreted with caution. Psychological abuse has been shown to be the most frequent characteristic of interpersonal violence dynamics, affecting women’s health as severely and significantly as other types of abuse [25,26]. However, the frequent co-occurrence of psychological aggression with physical violence, as well as difficulties in measurement, combine to reduce certainty with regard to the independent effects of psychological aggression on health. Some recent research showed that more men than women reported exposure to physical assault, while more women reported exposure to sexual coercion in the past year [37]. However, it should be mentioned that studies which found similar levels of IPV in women and men relied largely on past-year estimates or current partners, whereas studies assessing a longer time-frame or including previous partners tended to find dissimilar levels of violence [38]. The risk factors for IPV exposure in a cross-sectional population-based study in Sweden were the present relationship being of at least three years duration for men’s exposure, while a young age, lack of social support and being single constituted risk factors for women’s IPV exposure [37]. Compared with women who had never been abused, those reporting only psychological abuse were more likely to present negative mental health indicators in a study carried out in Spain [39].

Despite the abundant literature on the consequences of IPV on health, psychological abuse has rarely been considered a relevant health-related problem and has never been studied as such in Slovenia. After re-evaluating the prevalence data for past IPV abuse and showing an IPV exposure prevalence of approximately 17% as a valid estimate [4], it was decided to focus on the separate effects of psychological IPV on family medicine clinic attendees. This study aims to explore the differences between patients who were exposed to psychological IPV in the previous year and those who were not; to identify the health consequences and other patient characteristics associated with this type of IPV exposure; and to examine whether there were any specifically gender-related issues in family clinic attendees.

## Methods

### Participants and procedure

In January 2013, 90 general practitioners (GPs), i.e. family physicians who have finished four years of specialised training, who had already taken part in the 2012 IPV prevalence re-evaluation study [4], were invited to participate in this study. Sixty-four of them responded affirmatively (6.7% GPs listed in the Register of Family Medicine Doctors held in the Medical Chamber of Slovenia). They were provided with written instructions on the approach to the patients and data collection, i.e. the semi-structured interview forms and the medical charts review forms.

### The first phase: recruitment of patients

The first phase of data collection was carried out during the first week of March 2013, when the participating GPs, working in family practices all over the country, asked every fifth family practice attendee aged 18 years and above, regardless of gender, to participate in a study on quality of life. The eligibility criteria for this phase were age, the absence of dementia or even mild cognitive impairment, and the patients’ willingness to participate. The aim of the study was not explained, but the subjects were told that participation was not obligatory. Those willing to participate were scheduled for interview within two weeks. This phase of data collection ended either after 15 patients had been recruited, or on March 8, 2013, whichever was the earliest.

### The second phase: interviewing the patients

Of 960 invited patients, 689 interviewees came to family practices during the second phase, which lasted until the end of March. Since research has shown that disclosure of IPV violence is highly influenced by interviewer factors as well as privacy and the context of the interview [40], participating GPs were encouraged to revise the semi- structured interview form prior to interviewing patients, and to contact the coordinating researcher if in need of assistance or consultation.

**Figure 1:**
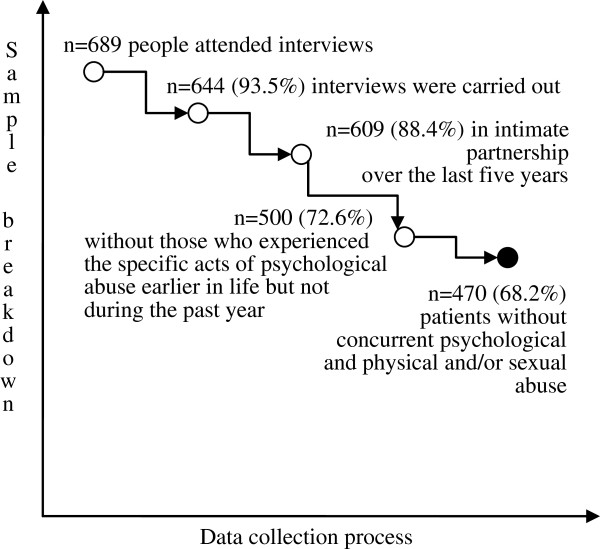
The data collection process.

At the beginning of the interview, the participants were told that exposure to IPV could be considered a serious quality of life issue in adults. After the topic was introduced, they were asked to sign a written consent form. Of the 689 patients, 45 decided against further participation; they were not asked to explain this decision. In total, 644 interviews were carried out and 609 patients declared they had had an intimate partner during the last five years. Those who had not been in an intimate relationship in the previous five year period were excluded from further analysis. A further 109 patients disclosed they had experienced specific acts of psychological abuse *earlier in life* but not *during the past year*, and another 30 people stated that they had been exposed to concurrent psychological and physical and/or sexual abuse. The latter group explained that aside from psychological abuse they endured behaviours such as hitting, slapping, kicking, pushing and/or being forced to engage in sexual activities against their will. As the study aimed to analyse solely psychological abuse in patients, those who had experienced psychological IPV during their lifetime but not in the past year were excluded from the analysis. Victims of multiple types of abuse were also excluded from the study, but were offered help and assistance. The drop-out data are presented in Figure 1.

### The third phase: auditing patients’ medical charts

After interviewing the patients, GPs reviewed their medical charts (n = 470) in the third phase of data collection. The Domestic Violence Exposure Medical Chart Check List from the 2009 study, described in detail elsewhere [2], was used for this purpose.

### Participants: study sample

The final sample consisted of 470 patients, who had been living in an intimate relationship during the past five years, and who had not had previous psychological abuse, or concurrent physical or sexual IPV exposure.

The National Medical Ethics Committee of the Republic of Slovenia approved the protocol of the study (document number 138/02/11).

### Measures

The semi-structured interview form consisted of several questions from the short form of A Domestic Violence Exposure Questionnaire, described by Kopcavar-Gucek et al. [3] and developed in previous studies in Slovenian primary care [2,41]. The questions gathered data on gender, age, number of children, marital status, number of divorces and place of residence. To identify psychological IPV adequately, more specific questions with comprehensive, behaviourally-defined descriptions of interpersonal violence events in closed questions were used in the semi-structured interview. The behavioural patterns of exposure to psychological violence were also inspired by the work of other authors [39,42]. This also aimed to avoid subjective understanding of the criteria of psychological violence in victims.

For better orientation in time the national holiday March 8, International Women’s Day, was used; this timeframe was introduced to patients with questions like: *What does March 8 mean to you personally? Do you acknowledge this holiday? What did you do this year? Are you able to recall the March 8 of last year?* The patients were told that the subsequent questions were to be considered within this timeframe and were then asked a series of questions: *Do you feel safe at home? Do you feel accepted, respected and loved in your intimate relationship? Have you been humiliated, subjected to threats, insult or intimidation, or in any way emotionally affected by your intimate partner? Does your partner talk down to you? Has he/she demeaned or insulted you or made you feel ashamed? Has he/she screamed or cursed at you? Has he/she threatened you with physical harm? If such a thing has happened, have you been thinking about doing something about it? Do you want me to help?* Patients were asked to specify whether this had happened in the 12 months preceding the survey, i.e. between March 8, 2012 and March 8, 2013.

These questions had several possible responses (i.e. *many times, sometimes, never, not last year, not this year* etc.). Participants were considered to have experienced “current psychological abuse” if they answered any question *many times* or *sometimes*. Others who answered to each question negatively were marked as negative for current abuse.

The second part of the survey, replicating the procedure of the 2009 study [2], was carried out by the GPs themselves after the interviews. Further information on the participating patients was gathered by auditing medical charts, including data on the patients’ wider life context. The physicians analyzed the patients’ medical charts and abstracted factors at both the personal level (i.e. *alcohol abuse*; *adult onset of depression*; *personality disorders*; *low education level*; *financial difficulties and instability* and *unemployment in patient*) and at a relationship level [i.e. *past experience of violence* (domestic violence exposure in primary family) reported by patient and marked in the medical chart; *disputes in the intimate partner relationship* (i.e. frequent differences of opinion or disagreements, struggles resulting from incompatible or opposing needs, values, demands and/or expectations), reported by patient and noted by the physician; and *dysfunctional family relationships* (e.g. male dominance in the family as a hardship), already discussed with the patient and marked by the physician], from the whole medical chart. Health-related associations with exposure to IPV were also listed and audited for the previous year (March 2012 to March 2013), and were later categorized into three groups: physical, sexual and reproductive, and psychological. Lastly, sick leave (in episodes and days), hospitalisation (in episodes and days), visits to family clinic and referrals to other clinical specialists in the past year were reviewed, noted and analysed. ‘Frequent’ among participating patients was defined as within the top 10 centiles in a time frame of one year for each characteristic (Table 1).

**Table 1:** Frequent use of health care services during the past year among participating patients

	**Top 10 centiles (a time frame of one year)**	**Psychological IPV exposure**		***P***_***ALL***_	**Psychological IPV exposure: women**		***P***_***WOMEN***_
		**No (N (%))**	**Yes (N (%))**		**No (N (%))**	**Yes (N (%))**	
Sick leave (episodes)	3 or more	44 (10.7)	4 (7.0)	0.491	30 (12.4)	4 (8.7)	0.621
Sick leave (days)	46 or more	43 (10.4)	4 (7.0)	0.636	23 (9.5)	4 (8.7)	1.000
Hospitalisation (episodes)	1 or more	30 (7.3)	6 (10.5)	0.422	13 (5.4)	6 (13.0)	0.095
Hospitalisation (days)	1 or more	30 (7.3)	6 (10.5)	0.422	13 (5.4)	6 (13.0)	0.095
Visits to family clinic	16 or more	40 (9.7)	4 (7.0)	0.634	28 (11.6)	4 (8.7)	0.798
Referrals to other clinical specialists	5 or more	37 (9.0)	7 (12.3)	0.464	21 (8.7)	5 (10.9)	0.582

### Data analysis

The sample data were presented as frequencies and percentages. Bivariate comparisons were made using the *χ*^2^ test or Fisher’s exact test. The analysis compared patients who reported having experienced any act of psychological violence in the past year (conceptualised as ‘current abuse’) with those who did not report any IPV (‘current’ *vs*. ‘never’) (Table 2). Demographic characteristics, the data obtained from patient medical charts for the time prior to the psychological IPV screening period, and current health status variables (see Table 3) were included in the modelling process, but not these without significant bivariate associations with past year psychological IPV exposure (dependent variable). Multivariate logistic regression was used to model those factors associated with past year psychological IPV exposure in the total sample and separately for women only (Tables 4 and 5).

**Table 2:** Demographics and health services use in patients

	**Total N (%)**	**IPV exposure N (%)**		**p**
	**n = 470**	**No n = 413**	**Yes n = 57**	
**Gender**				**0.001**
Male	182 (38.7)	171 (41.4)	11 (19.3)	
Female	288 (61.3)	242 (58.6)	46 (80.7)	
**Age**				0.809
35 years or less	139 (29.6)	124 (30.0)	15 (29.6)	
36–64 years	254 (54.0)	221 (53.5)	33 (57.9)	
65 years and more	77 (16.4)	68 (16.5)	9 (15.8)	
**Present marital status**				0.471
Married or living in an intimate relationship	352 (74.9)	306 (74.1)	46 (80.7)	
Widowed	54 (11.5)	50 (12.1)	4 (7.0)	
Single	64 (13.6)	57 (13.8)	7 (12.3)	
**Age of intimate partner**				**0.028**
The same or younger	334 (71.1)	301 (72.9)	33 (57.9)	
Older	136 (28.9)	112 (27.1)	24 (42.1)	
**Parenting (children)**				0.252
No	126 (26.8)	114 (27.6)	12 (21.1)	
Single child	117 (24.9)	98 (23.7)	19 (33.3)	
Two children or more	227 (48.3)	201 (48.7)	26 (45.6)	
**Divorce in the past**				0.591
Yes	88 (18.7)	76 (18.4)	12 (21.1)	
No	382 (81.3)	337 (81.6)	45 (78.9)	
**Place of residence**				1.000
Rural	53 (11.3)	47 (11.4)	6 (10.5)	
Urban	417 (88.7)	366 (88.6)	51 (89.5)	
**Employment status**				**0.010**
Regularly employed	273 (58.1)	248 (60.0)	25 (43.9)	
Retired	119 (25.3)	104 (25.2)	15 (26.3)	
Unemployed or working part time	78 (16.6)	61 (14.8)	17 (29.8)	
**Level of education**				0.416
Elementary school	52 (11.1)	48 (11.6)	4 (7.0)	
High school	203 (43.2)	180 (43.6)	23 (40.4)	
College or higher	215 (45.7)	185 (44.8)	30 (52.6)	
**Monthly income per family member**				0.670
Under average	266 (56.6)	232 (56.2)	34 (59.6)	
Average or above	204 (43.4)	181 (43.8)	23 (40.4)	
**Financial support provided by state**				0.512
Yes	115 (24.5)	99 (24.0)	16 (28.1)	
No	355 (75.5)	314 (76.0)	41 (71.9)	
**The length of intimate partnership**				0.078
5 years or less	165 (35.1)	151 (36.6)	14 (24.6)	
6 years or more	305 (64.9)	262 (63.4)	53 (75.4)	
**Alcohol or drug consumption in the family**				**0.028**
Yes	136 (28.9)	112 (27.1)	24 (42.1)	
No	334 (71.1)	301 (72.9)	33 (57.9)	
Sick leave in the past year (episodes) – M ± SD	1.1 ± 2.3	1.1 ± 2.1	1.2 ± 3.4	0.114
Sick leave in the past year (days) – M ± SD	22.8 ± 63.2	23.5 ± 63.6	16.4 ± 59.8	0.091
Hospitalisation in the past year (episodes) – M ± SD	0.1 ± 0.4	0.1 ± 0.4	0.1 ± 0.4	0.413
Hospitalisation in the past year (days) – M ± SD	1.0 ± 5.0	1.0 ± 5.3	0.9 ± 2.9	0.418
Visits to family clinic in the past year – M ± SD	8.2 ± 5.5	8.3 ± 5.5	7.6 ± 5.1	0.465
Referrals to other clinical specialists in the past year – M ± SD	2.0 ± 2.2	2.0 ± 2.2	2.2 ± 2.3	0.958
Age – M ± SD	47.4 ± 16.1	47.4 ± 16.2	47.8 ± 15.6	0.795

Chi-square or Fisher’s exact test; Mann-Whitney’s *U* test for continuous data.

**Table 3:** Patients’ medical charts review summary

	**No IPV exposure n = 413**	**IPV exposure n = 57**	p*
**Physical status**	381 (92.3%)	54 (94.7%)	0.787
Injuries: head, thoracic and abdominal compartment	90 (21.8%)	12 (21.1%)	1.000
Scratches and bruises	162 (39.2%)	24 (42.1%)	0.668
Chronic pain syndrome	324 (78.5%)	47 (82.5%)	0.604
Incapacity to work	87 (21.1%)	15 (26.3%)	0.392
Muscle inflammations	176 (42.6%)	35 (61.4%)	0.010
Bone fractures	92 (22.3%)	13 (22.8%)	1.000
Gastrointestinal disorders	256 (62.0%)	43 (75.4%)	0.056
Irregularities in bowel functioning	187 (45.3%)	28 (49.1%)	0.671
Lacerations and cuts	134 (32.4%)	18 (31.6%)	1.000
Eye injuries	23 (5.6%)	2 (3.5%)	0.755
Reduced physical functioning	41 (9.9%)	7 (12.3%)	0.639
**Sexual and reproductive status**	201 (48.7%)	38 (66.7%)	0.011
Gynaecological disorders, inflammations	186 (45.0%)	36 (63.2%)	0.011
Infertility	25 (6.1%)	4 (7.0%)	0.768
Complicated pregnancies/spontaneous abortions	55 (13.3%)	14 (24.6%)	0.043
Sexual dysfunctions	14 (3.4%)	3 (3.5%)	1.000
Sexually transmitted diseases including HIV/AIDS	9 (2.2%)	2 (3.5%)	0.631
Unplanned/unwanted pregnancies	3 (0.7%)	1 (1.8%)	0.405
**Psychological and behavioural status**	346 (83.8%)	57 (100.0%)	<0.001
Abuse of alcohol and drugs	26 (6.3%)	3 (5.3%)	1.000
The onset of depression and/or generalised anxiety disorder	272 (65.9%)	51 (89.5%)	<0.001
Eating and sleeping disorders	151 (36.3%)	29 (50.9%)	0.042
Low self-esteem	173 (41.9%)	39 (68.4%)	<0.001
Phobias and panic attacks	144 (34.9%)	33 (57.9%)	0.001
Physical inactivity	149 (36.1%)	23 (40.4%)	0.559
Post-traumatic stress disorder	148 (35.8%)	33 (57.9%)	0.002
Psychosomatic disorders	292 (70.7%)	50 (87.7%)	0.007
Smoking	90 (21.8%)	14 (24.6%)	0.613
Suicidal behaviour and self-harm	4 (1.0%)	1 (1.8%)	0.478
Unsafe sexual behaviour	1 (0.2%)	0 (0.0%)	1.000

*Fisher’s exact test (instead of the chi-square for 2x2 tables).

**Table 4:** Factors associated with intimate partner violence exposure in the previous year

	**No IPV n = 413**	**Psych IPV n = 57**	**cOR (95% CI)**	**p**	**aOR (95% CI)**	**p**
Female gender	242 (58.6)	46 (80.7)	2.96 (1.49–5.87)	0.002	2.44 (0.96–6.18)	0.060
Marital status						
Married or living in intimate partnership	306 (74.1)	46 (80.7)	1.00 (reference)		1.00 (reference)	
Widowed	50 (12.1)	4 (7.0)	0.53 (0.18–1.54)	0.245	0.90 (0.15–5.32)	0.905
Single	57 (13.8)	7 (12.3)	0.82 (0.35–1.90)	0.639	1.06 (0.19–5.85)	0.949
Divorce in the past	76 (18.4)	12 (21.1)	1.18 (0.60–2.34)	0.631	0.69 (0.25–1.88)	0.468
Living in urban setting	366 (88.6)	51 (89.5)	1.09 (0.44–2.68)	0.849	1.09 (0.35–3.37)	0.877
Employment status						
Regularly employed	248 (60.0)	25 (43.9)	1.00 (reference		1.00 (reference)	
Retired	104 (25.2)	15 (26.3)	1.43 (0.73–2.82)	0.302	2.18 (0.66–7.13)	0.199
Unemployed or working part time	61 (14.8)	17 (29.8)	2.77 (1.41–5.44)	0.003	5.82 (2.09–16.17)	0.001
Level of education						
Elementary school	48 (11.6)	4 (7.0)	1.00 (reference		1.00 (reference)	
High school	180 (43.6)	23 (40.4)	1.53 (0.51–4.65)	0.450	2.30 (0.58–9.14)	0.237
College degree or more	185 (44.8)	30 (52.6)	1.95 (0.65–5.79)	0.231	4.78 (1.09–20.96)	0.038
Below-average monthly income per family member	232 (56.2)	34 (59.6)	1.15 (0.66–2.03)	0.620	0.95 (0.39–2.28)	0.904
Financial support provided by state	99 (24.0)	16 (28.1)	1.24 (0.67–2.30)	0.500	1.35 (0.55–3.34)	0.511
The length of intimate relationship ≥6 years	262 (63.4)	43 (75.4)	1.77 (0.94–3.34)	0.078	4.25 (1.01–17.85)	0.048
Alcohol or drug consumption in the family	112 (27.1)	24 (42.1)	1.96 (1.11–3.45)	0.021	1.55 (0.71–3.37)	0.272
Age						
35 years or less	124 (30.0)	15 (26.3)	1.00 (reference		1.00 (reference)	
36–64 years	221 (53.5)	33 (57.9)	1.23 (0.65–2.36)	0.525	0.92 (0.31–2.74)	0.885
65 years or more	68 (16.5)	9 (15.8)	1.09 (0.46–2.63)	0.841	1.03 (0.19–5.49)	0.976
Parenting						
No	114 (27.6)	12 (21.1)	1.00 (reference)		1.00 (reference)	
One child	98 (23.7)	19 (33.3)	1.84 (0.85–3.98)	0.121	0.68 (0.19–2.40)	0.547
Two children or more	201 (48.7)	26 (45.6)	1.23 (0.60–2.53)	0.576	0.51 (0.14–1.88)	0.315
Older intimate partner	112 (27.1)	24 (42.1)	1.96 (1.11–3.45)	0.021	1.88 (0.80–4.42)	0.150
Patient’s MCR: personality disorders	26 (6.3)	7 (12.3)	2.08 (0.86–5.05)	0.104	0.88 (0.29–2.72)	0.828
Patient’s MCR: domestic violence exposure in primary family	103 (24.9)	23 (40.4)	2.04 (1.15–3.62)	0.015	1.35 (0.60–3.06)	0.466
Patient’s MCR: history of disputes in intimate relationship	104 (25.2)	46 (80.7)	12.43 6.21–24.88)	<0.001	13.85 (5.72–33.55)	<0.001
Patient’s MCR: financial difficulties and instability	227 (55.0)	44 (77.2)	2.77 (1.45–5.30)	0.002	1.22 (0.46–3.27)	0.686
Patient’s MCR: dysfunctional family relations	189 (45.8)	42 (73.7)	3.32 (1.78–6.17)	<0.001	1.37 (0.59–3.18)	0.467
Patient’s MCR: history of unemployment	130 (31.5)	26 (45.6)	1.82 (1.04–3.20)	0.035	0.66 (0.26–1.68)	0.386
Patient’s MCR: muscular inflammations	176 (42.6)	35 (61.4)	2.14 (1.21–3.78)	0.009	1.18 (0.56–2.52)	0.662
Patient’s MCR: gynaecological disorders, inflammations	137 (33.2)	34 (59.6)	2.98 (1.69–5.25)	<0.001	0.98 (0.43–2.24)	0.959
Patient’s MCR: complications during pregnancy/spontaneous abortions	55 (13.3)	13 (22.8)	1.92 (1.09–4.13)	0.060	1.07 (0.36–3.17)	0.909
Patient’s MCR: the onset of depression and/or generalised anxiety disorder	272 (65.9)	51 (89.5)	4.41 (1.85–10.52)	0.001	1.39 (0.37–5.23)	0.630
Patient’s MCR: eating and sleeping disorders	151 (36.6)	29 (50.9)	1.80 (1.03–3.14)	0.039	0.93 (0.42–2.03)	0.849
Patient’s MCR: phobias and panic attacks	144 (34.9)	33 (57.9)	2.57 (1.46–4.51)	0.001	2.30 (0.97–5.44)	0.057
Patient’s MCR: low level of self-esteem: Expressing shame and guilt	173 (41.9)	39 (68.4)	3.01 (1.66–5.43)	<0.001	1.32 (0.53–3.31)	0.550
Patient’s MCR: post-traumatic stress disorder	148 (35.8)	33 (57.9)	2.46 (1.40–4.32)	0.002	0.80 (0.33–1.97)	0.632
Patient’s MCR: psychosomatic disorders	292 (70.7)	50 (87.7)	2.96 (1.31–6.71)	0.009	0.63 (0.19–2.10)	0.451

cOR: crude odds ratio.

aOR: adjusted odds ratio – adjusted for age, gender, and all other independent variables in the table.

Patient’s MCR: Patient’s Medical Chart Review.

A Logistic Regression Model: *χ*^2^ = 114.112, df = 33, p < 0.001, Nagelkerke R^2^ = 0.413.

**Table 5:** Factors associated with intimate partner violence exposure in the previous year in female patients

	**No IPV n = 242**	**Psych IPV n = 46**	**cOR (95% CI)**	**p**	**aOR (95% CI)**	**p**
Female gender				-		-
Marital status						
Married or living in intimate partnership	172 (71.1)	38 (82.6)	1.00 (reference)		1.00 (reference)	
Widowed	38 (15.7)	4 (8.7)	0.48 (0.16–1.42)	0.182	0.60 (0.08–4.31)	0.613
Single	32 (13.2)	4 (8.7)	0.57 (0.19–1.70)	0.309	0.40 (0.05–3.02)	0.373
Divorce in the past	50 (20.7)	12 (26.1)	1.36 (0.65–2.81)	0.413	1.06 (0.31–3.56)	0.929
Living in urban setting	217 (89.7)	41 (89.1)	0.95 (0.34–2.61)	0.913	0.84 (0.22–3.24)	0.805
Employment status						
Regularly employed	151 (62.4)	20 (43.5)	1.00 (reference)		1.00 (reference)	
Retired	63 (26.0)	12 (26.1)	1.44 (0.66–3.12)	0.357	1.71 (0.41–7.07)	0.458
Unemployed or working part time	28 (11.6)	14 (30.4)	3.78 (1.71–8.34)	0.001	7.82 (2.20–27.85)	0.002
Level of education						
Elementary school	27 (11.2)	4 (8.7)	1.00 (reference)		1.00 (reference)	
High school	103 (42.6)	20 (43.5)	1.31 (0.41–4.16)	0.646	1.55 (0.31–7.69)	0.593
College degree or more	112 (46.3)	22 (47.8)	1.33 (0.42–4.17)	0.629	2.02 (0.38–10.80)	0.411
Below-average monthly income per family member	137 (56.6)	29 (63.0)	1.31 (0.68–2.51)	0.419	1.34 (0.47–3.87)	0.587
Financial support provided by state	62 (25.6)	11 (23.9)	0.91 (0.44–1.91)	0.807	1.13 (0.37–3.51)	0.829
The length of intimate relationship ≥6 years	142 (58.7)	35 (76.1)	2.24 (1.09–4.62)	0.029	2.75 (0.63–12.08)	0.181
Alcohol or drug consumption in the family	65 (26.9)	22 (47.8)	2.50 (1.31–4.76)	0.005	2.33 (0.92–5.94)	0.075
Age						
35 years or less	79 (32.6)	11 (23.9)	1.00 (reference)		1.00 (reference)	
36–64 years	125 (51.7)	28 (60.9)	1.61 (0.76–3.41)	0.215	1.32 (0.36–4.87)	0.673
65 years or more	38 (15.7)	7 (15.2)	1.32 (0.48–3.68)	0.592	1.06 (0.13–8.51)	0.956
Parenting						
No	60 (24.8)	8 (17.4)	1.00 (reference)		1.00 (reference)	
One child	67 (27.7)	17 (37.0)	1.90 (0.77–4.73)	0.166	0.80 (0.16–4.01)	0.785
Two children or more	115 (47.5)	21 (45.7)	1.37 (0.57–3.28)	0.480	0.51 (0.10–2.64)	0.425
Older intimate partner	101 (41.7)	22 (47.8)	1.28 (0.68–2.41)	0.445	1.35 (0.53–3.46)	0.532
Patient’s MCR: personality disorders	13 (5.4)	5 (10.9)	2.15 (0.73–6.35)	0.167	0.85 (0.20–3.59)	0.824
Patient’s MCR: domestic violence exposure in primary family	67 (27.7)	17 (37.0)	1.53 (0.79–2.97)	0.207	0.82 (0.31–2.19)	0.690
Patient’s MCR: history of disputes in intimate relationship	64 (26.4)	37 (80.4)	11.43 (5.23–25.01)	<0.001	9.12 (3.34–24.89)	<0.001
Patient’s MCR: financial difficulties and instability	128 (52.9)	37 (80.4)	3.66 (1.69–7.92)	0.001	1.45 (0.41–5.09)	0.567
Patient’s MCR: dysfunctional family relations	113 (46.7)	36 (78.3)	4.11 (1.95–8.66)	<0.001	2.35 (0.78–7.04)	0.127
Patient’s MCR: history of unemployment	70 (28.9)	20 (43.5)	1.89 (0.99–3.61)	0.053	0.44 (0.15–1.30)	0.137
Patient’s MCR: muscular inflammations	101 (41.7)	28 (60.9)	2.17 (1.14–4.14)	0.018	1.20 (0.51–2.85)	0.673
Patient’s MCR: gynaecological disorders. inflammations	137 (56.6)	34 (73.9)	2.17 (1.07–4.40)	0.031	1.67 (0.58–4.80)	0.339
Patient’s MCR: complications during pregnancy/spontaneous abortions	55 (22.7)	13 (28.3)	1.34 (0.66–2.72)	0.419	0.61 (0.18–2.12)	0.437
Patient’s MCR: the onset of depression and/or generalised anxiety disorder	160 (66.1)	42 (91.3)	5.38 (1.87–15.53)	0.002	1.25 (0.22–7.01)	0.797
Patient’s MCR: eating and sleeping disorders	93 (38.4)	24 (52.2)	1.75 (0.93–3.29)	0.084	1.13 (0.45–2.81)	0.792
Patient’s MCR: phobias and panic attacks	84 (34.7)	26 (56.5)	2.45 (1.29–4.64)	0.006	1.59 (0.59–4.29)	0.360
Patient’s MCR: low level of self-esteem: expressing shame and guilt	100 (41.3)	33 (71.7)	3.61 (1.81–7.19)	<0.001	1.27 (0.42–3.84)	0.667
Patient’s MCR: post-traumatic stress disorder	85 (35.1)	27 (58.7)	2.63 (1.38–5.00)	0.003	0.83 (0.28–2.43)	0.731
Patient’s MCR: psychosomatic disorders	174 (71.9)	42 (91.3)	4.10 (1.42–11.88)	0.009	0.82 (0.18–3.65)	0.793

cOR: crude odds ratio.

aOR: adjusted odds ratio – adjusted for age and all other independent variables in the table.

Patient’s MCR: Patient’s Medical Chart Review.

A Logistic Regression Model: *χ*^2^ = 83.530, df = 32, p < 0.001, Nagelkerke R^2^ = 0.431.

With the aim of avoiding conceptual overlapping between the independent variable *disputes in intimate relationship* and the dependent variable, another regression modelling was performed without this independent variable, which had been earlier identified as a powerful risk factor for psychological IPV exposure. Similarly, an additional regression modelling was made for female patients without the independent variable *disputes in intimate relationship*.

The results were presented by crude and adjusted odds ratios with 95% confidence intervals. Statistical analysis was performed by IBM SPSS 20.0 software (IBM Corp., Armonk, NY, USA), and *P* < 0.05 was set as the level of statistical significance.

## Results

Of the sample, 12.1% (n = 57) people had been exposed to psychological IPV in the previous year (46 women and 11 men).

### Demographic characteristics of patients

The average age of all participants (n = 470) was 47.4 ± 16.1 years; the average age of men (n = 182) was 48.2 ± 15.8 years and of women (n = 288) 46.9 ± 16.4 years (p = 0.279). The average age of patients without experience of psychological IPV (n = 413) was 47.4 ± 16.2 years; men (n = 171) 48.6 ± 15.6 years and women (n = 242) 46.5 ± 16.6 (p = 0.117). People who had been exposed to psychological abuse were 47.8 ± 15.6 years old; of them the men were 42.7 ± 17.7 and the women 49.0 ± 15.1 years old, the latter being seemingly but not significantly older (p = 0.210).

More demographic characteristics of the sample are presented in Table 2.

### Frequent use of health care services during the past year among participating patients

There were no statistically significant differences found between people exposed to psychological IPV and those who did not report such experiences (Table 1).

### Patients’ medical charts review summary

In Table 3, the summary of patients’ medical charts is presented. Statistically significant differences were identified between the patients exposed and those not exposed to psychological IPV, with regard to muscle inflammations (p = 0.010), sexual and reproductive status in general (p = 0.011), specifically gynaecological disorders and inflammations (p = 0.011), complicated pregnancies/spontaneous abortions (p = 0.043), psychological and behavioural status in general (p <0.001), including the onset of depression and/or generalised anxiety disorder (p <0.001), eating and sleeping disorders (p = 0.042), low self-esteem (p < 0.001), phobias and panic attacks (p = 0.001), post-traumatic stress disorder (p = 0.002) and psychosomatic disorders (p = 0.007), all assessed or diagnosed by practising GPs in the year prior to the survey.

### Associations between psychological intimate partner violence exposure and bio-psycho-social characteristics in patients: logistic regression modelling

In the regression modelling process, the associations between psychological IPV and the characteristics of patients were explored. Employment status (i.e. unemployed or working part-time) (aOR 5.82, 95% CI 2.09-16.17, p = 0.001), level of education, i.e. college degree or more (aOR 4.78, 95% CI 1.09-20.96, p = 0.038), the length of intimate relationship ≥ 6 years (aOR 4.25, 95% CI 1.01-17.85, p = 0.048) and a history of disputes in the intimate relationship already noted in the patient’s medical chart (aOR 13.85, 95% CI 5.72-33.55, p <0.001) were identified as risk factors, explaining 41% of the variance (Nagelkerke R^2^ = 0.413, p < 0.001). More results are presented in Table 4.

In another regression modelling performed without independent variable *disputes in intimate relationship*, 27% of the variance was explained (*χ*^2^ = 73.156, df = 32, p < 0.001, Nagelkerke R^2^ = 0.276), with employment status (i.e. unemployed or working part-time) (aOR 5.57, 95% CI 2.23-13.91, p < 0.001) and level of education, i.e. college degree or more (aOR 4.58, 95% CI 1.22-17.20, p = 0.024) identified as the sole risk factors. Since the other independent variables were the same as presented in Table 4, the other results of this additional regression analysis are not presented.

### Associations between psychological intimate partner violence exposure and bio-psycho-social characteristics of female patients: logistic regression modelling

Table 5 presents a logistic regression model of psychological IPV exposure and its associations in female patients. Employment status (i.e. unemployed or working part-time) (aOR 7.82, 95% CI 2.20-27.85, p = 0.002) and a history of disputes in the intimate relationship identified by the GP in the patient’s medical chart review (aOR 9.12, 95% CI 3.34-24.89, p < 0.001) increased the odds of exposure to psychological IPV in female patients, with regression modelling explaining 43% of the variance (Nagelkerke R^2^ = 0.431, p < 0.001).

In an additional regression modelling made for female patients without the independent variable *disputes in intimate relationship*, 33% of the variance were explained (*χ*^2^ = 61.712, df = 31, p = 0.001, Nagelkerke R^2^ = 0.330), and the only risk factor identified was the patient’s unemployment or working part-time (aOR 10.48, 95% CI 3.23-33.97, p < 0.001). All other independent variables were not significantly associated with psychological IPV exposure, so this additional regression modelling is not presented in a table.

## Discussion

The prevalence of psychological IPV of 12.1% during the past year was similar to the prevalence of 10.3% found in the 2012 re-evaluation study of Slovenian family medicine attendees [4], regardless of different time period in question (a year *vs.* five years). As stated by Feder et al. [43], asking people about a longer period of time or recent experience can both be potentially problematic; recall bias may be present in responses about a longer period of time, as in the 2012 study [4], while participants in this study, focused on past year violence, might have had insufficient time to acknowledge or identify their abusive experiences as such. It would be fair to conclude that in psychological IPV exposure a prevalence of about 10% is a correct estimation, although it can still be re-evaluated. This prevalence is also concordant with the findings in the WHO multi-country observational study on women’s health and domestic violence [44], which reported that between 4%-54% of respondents were exposed to IPV in the year prior to the survey. It was noted that sampling and the time period in question might have led to differences with respect to the gender symmetry of IPV [45,46]. Our findings showed differences in psychological IPV between male and female patients (6% *vs*. 16%) (Table 2). It has been suggested that men generally experience less threatening and less severe forms of IPV, so they may not consider it particularly salient to remember later in life; similarly, given that women are generally exposed to more severe forms of IPV with higher levels of physical injury, coercive control and fear, they may be more likely to report such violence later in life [46]. Since we explored patients’ experiences for only one year previous to the data collection, we do not believe that it affected recall in male participants.

### Associations between psychological intimate partner violence exposure and health status and the use of health care services

Given that psychological abuse often precedes physical abuse [27], and has been found to be as strongly associated with the majority of adverse health outcomes as physical IPV [25], the findings of this study could serve as a useful tool for GPs to aid improved detection of psychological IPV and proper early intervention, although people exposed to psychological IPV have not been shown to use health care services more (Tables 1 and 2).

The incidence of several, mostly psychological and behavioural status-related conditions (e.g. depression and anxiety, eating and sleeping disorders, low self-esteem, phobias and panic attacks, post-traumatic stress disorder (PTSD), psychosomatic disorders), was found to be significantly higher in psychologically abused patients’ medical charts review (Table 3), which is concordant with the findings of other authors [47-50]. Psychological IPV was found to be as detrimental as physical IPV in terms of depressive symptoms [51], and also to be a significant predictor of higher levels of IPV-related depression [52]. Women reporting IPV have been shown to be significantly more likely to experience a greater degree of depressive symptoms and functional impairment with lower self-esteem and life satisfaction at five-year follow up [53]. A history of IPV was also found to be positively associated with increased incidence of PTSD symptoms, PTSD diagnoses [54,55], and increased levels of anxiety in women [47,54,56,57]. An association between the severity of anxiety symptoms and co-occurring depression has also been reported, with the severity of anxiety being higher in abused women with depressive symptoms [51]. As expected, gynaecological disorders were more prevalent in the emotionally abused participants of this study (Table 3), since gynaecological symptoms were also reported to be associated with a history of IPV in many other studies [58-60].

However, all of these health conditions were not significantly associated with psychological IPV exposure in multivariate modelling procedures used to partition the variance in a wide variety of indicators of participants’ experiences (Tables 4 and 5). When discussing these results, adjusted odds ratios (aOR) between 0.95 > OR < 1.05 (p < 0.05) were considered as indicative of no association and aORs of 1.05 or greater (p < 0.05) as risk factors for psychological IPV exposure. In case of aORs of 0.95 or less, with p < 0.05 set as the level of statistical significance, we would have been discussing protective factors for IPV. However, there were none identified. The term *risk factor* is used loosely to indicate the direction of association with IPV rather than to imply causality, as we have been analysing mainly cross-sectional data.

Regardless of gender, most variation was associated with employment status, level of education, an intimate relationship of longer than six years, and a history of disputes in the intimate relationship (Table 4), while in women, unemployment and a history of disputes in the intimate relationship, identified by the GP in the patient’s medical chart review, were also identified as risk factors for psychological IPV exposure (Table 5). Apart from a college degree, identified as a risk factor in the whole sample, we believe that unemployment, which increases the odds of being exposed to psychological IPV by over five times, could also be considered as a risk factor for the onset of depression and/or anxiety, as diagnosed in 89.5% (p < 0.001) of IPV-exposed subjects (Table 3). Otherwise, apart from a genetic predisposition and stressful life events, the known risk factor for depression is a lower socioeconomic status (unemployment rate, lower education, poverty) [61,62]. The literature review by Kessler et al. [63] is consistent in showing a strong co-morbidity between general anxiety disorder and adult onset of depression, which was the reason we constructed a single variable for the analysis, i.e. *The onset of depression and/or generalised anxiety disorder* in the medical history (Tables 3, 4 and 5). In Slovenia, Klemenc Ketis et al. [64] reported a prevalence of 15.2% patients with depression in the adult population aged between 18 and 64 years. The incidence of depression and/or anxiety in our sample was apparently higher; however, it needs to be further tested in IPV exposed patients at least once more before any valid conclusions can be drawn.

### The harmful impact of employment status

In the first quarter of 2012, at the time period in question for this study, the registered unemployment rate, defined as the percentage of unemployed people in the labour force in Slovenia, was 12.3% [65]. In the last four years, the number of employed people in the country was reduced by 70000, while the population of unemployed has grown by 39000; of these, approximately one half was younger than 35 years [65]. In our study, 16.6% of people were unemployed or working part-time (Table 2), and the percentage is significantly higher (p = 0.010) in IPV-exposed people (29.8%) compared to those not exposed (14.8%); this data obviously shows the unemployment of the study participants as being above the national rate. The official rate of unemployment in this country has been growing from 10.7% in 2010 to 13.6% in 2013, and the trend is expected to continue [65], which should be considered a threat to the well-being of the general population in Slovenia. Unemployment in women and in general was identified as a powerful risk factor for psychological IPV exposure in this study, presumably associated with poverty or at least with the threat thereof. Violence was reported to be frequently used with the aim of resolving conflicts caused by poverty [23].

### Intimate partnership characteristics and educational attainment as risk factors

A history of disputes in the intimate relationship was shown to be the most powerful risk factor (Tables 4 and 5). As stated by Jewkes [66], relationships full of conflict, especially those in which conflicts occur about finance, jealousy, and women’s gender role transgressions, are more likely to be violent. Educationally, economically, and socially empowered women were found to be the most protected, the relation between empowerment and risk of IPV being presented as non-linear [66]. Contrary to this, in our study sample, a college degree increased the odds of psychological abuse (Table 4). On 1 January 2012, 19% of Slovenian citizens had tertiary education, i.e. a college degree or higher. Among the employed population, 29% had attained this level of education, while the proportion of highly educated people in the unemployed population was 14% at that time [67]. Of the participants in this study (Table 2), 45.7% people had attained a college degree or higher; in those exposed to IPV this rate was 52.6%, while among those not exposed it was 44.8% (p = 0.416). This rate is apparently well above the national average. Aside from formal marriage, the WHO multi-country study on women’s health and domestic violence showed secondary education and a high socio-economic status to be protective factors for IPV exposure [68]. The increased likelihood of better educated participants being exposed to psychological abuse in this study (Table 4) could be explained by their perception and sensibility toward IPV-related behaviour. Whether or not it is reasonable to expect better-educated people to be less tolerant of psychological IPV due to their increased knowledge, norms, values and expectations, is yet to be discovered. Further exploration, probably based on a qualitative approach, would be needed to test this hypothesis.

Since conflicts and disputes in the intimate relationship could have been perceived and interpreted by the patients as psychological IPV itself, additional modelling was performed and employment status identified as a risk factor in both the whole sample and in women only. Aside from the risk factors already discussed, the length of the intimate relationship (≥ 6 years – Table 4) increased the odds of participants being exposed to psychological IPV, similarly to other findings [37]. However the same was not identified in female patients analysed separately, and although somehow understandable, it needs further verification.

### Implications for family practice and future research

Generally, the sample in this study could not be considered as representative of a family medicine attendees’ population. In comparison to a representative sample of Slovenian family practice attendees [69], in our sample there were more women (61.3% *vs.* 54.8%), the mean age was slightly younger (47.4 ± 16.1 years *vs.* 51.7 ± 19.0), and level of education higher (45.7% *vs.* 11.3% people with college degree or above, 11.1% *vs*. 41.0% with elementary school).

Our results failed to demonstrate that psychological IPV alone is highly detrimental to patients’ health, as has been suggested by others [54]. Possible explanation might have been difficulties in managing ethical dilemmas in Slovenian family medicine, given that Klemenc-Ketis et al. [70] found one of the most difficult ethical issues for GPs to be suspicion of physical abuse, sexual abuse, or other criminal behaviour exposure in patients. However, we believe one of the main reasons for our findings was the time period, i.e. *current exposure*. Moreover, the results are important, as psychological IPV is often still considered a minor type of violence in Slovenia, and consequently receives less attention than physical IPV from clinicians, lawyers, policy makers, researchers and the female victims themselves. Thus, exposure to psychological IPV alone should no longer be considered a minor type of IPV in family medicine practices. More importantly, as stated by Blasco-Ros et al. [54], psychological IPV alone should be considered as less likely to cease than physical IPV or concurrent psychological and physical IPV. Therefore our advice for GPs would be to consider the possibility of exposure to psychological IPV alone in patients who have persistent complaints regarding their psychological and behavioural status (Table 3).

The cross-sectional survey design is inherently limited and, together with reliance on self-reported data, raises questions about the potential for method variance to account for our findings. However, the phenomenon being studied could have been assessed only by asking patients to report their experience or perception, and it was advantageous that this research design incorporated medical records to obtain the exact health related data (marked as *Patient’s Medical Chart Review* – see Tables 3, 4 and 5). Aside from the self-reported data, several characteristics were followed through the longer period of time reviewed by the GPs in the patients’ medical history, which we consider mitigated the potential effects of method variance. In the modelling process, data obtained from patients’ medical charts for the time prior to psychological IPV screening period and current health status variables were included. This allowed us to explore the effects of early-life characteristics and experiences. Although there were a few factors identified as being associated with psychological IPV exposure, and none of them could be considered as a medical condition, the advantage of this study is the partition of the explained variance (41% and 43% respectively). Our finding that health status data from the previous year were not associated with the current psychological IPV exposure suggests the importance of taking this time frame into account when assessing psychological IPV-related health conditions. Prospective studies using clear diagnostic criteria and measures, as well as in-depth, qualitative studies, would be beneficial for extending and deepening our understanding of bio-psycho-social patterns in psychological IPV-exposed patients. We believe that further research should also focus on a longer period of time in order to get more concise characteristics and better grounds for preventive action planning at the societal level and also in the field of family medicine in Slovenia.

### Limitations to the study

Data on each GP’s drop-out rate (i.e. the response rate from the first to the second phase of recruitment of patients) was not analysed. Of 960 scheduled patients, 689 (71.8%) attended the interview. One of the reasons for this first drop-out could have been eligibility criteria at the first phase of data collection, i.e. age, the absence of dementia or even mild cognitive impairment, and patients’ willingness to participate. Since patients coming for administrative purposes, i.e. chronic patients coming for prescriptions and patients requiring sick leave forms, were also included, their need for health care services could have been fulfilled by getting what they had come for, so later there was no intrinsic motivation for them to attend the scheduled interviews. We believe that the recruitment capacity of GPs could also have been associated with the quality of their relationships with the patients.

The question of the validity of GPs’ assessment of psychological and behavioural status and its components (Tables 3, 4 and 5) should also be raised. For the time being, it remains unclear whether or not the onset of depression and/or generalised anxiety disorder, eating and sleeping disorders, phobias and panic attacks, post-traumatic stress disorder and psychosomatic disorders were diagnosed as meeting all relevant criteria and guidelines.

Finally, the already-mentioned disadvantage that our findings are not based on a representative sample of family practice attendees in Slovenia should be considered a serious limitation; therefore the identified risk factors could serve as relatively valid guidance for family physicians only in middle-aged, better educated and predominantly female patients.

## Conclusions

The prevalence data of psychological IPV exposure in 12.1% of people are concordant with our previous findings in Slovenia. In the sample, the predominance of better-educated people might have been associated with lower tolerance towards psychological IPV. Unemployment in patients should be taken seriously in family medicine attendees, if GPs want to recognise psychological IPV and intervene effectively in individual cases. The state of economical and societal affairs in a country where the unemployment rate is still growing must be acknowledged, and GPs should strengthen their role as their patients’ advocates in the broadest meaning of bio-psycho-social well-being.

The results of this study, although aimed at exploring gender-related patterns of psychological IPV, warn of the possible damaging impact of employment status, based on prospective data in patients’ medical charts. It could be of utmost importance since psychological abuse often precedes other forms of interpersonal violence.

## Abbreviations

GP: A family physician who has finished four years of specialised training; cOR: Crude odds ratio; aOR: Adjusted odds ratio; Patient’s MCR: Patient’s Medical Chart Review.

## Competing interests

The authors declare that they have no competing interests.

## Authors’ contributions

PS conceived the study and drafted the manuscript. IS participated in interpretation and helped to draft the manuscript. NKG carried out the execution of the study. All the authors read and approved the final manuscript.

## Authors’ information

PS: Ph.D. Clinical Psychology, Senior Researcher and Associate Professor at the Department of Family Medicine. IS: Ph.D. Family Medicine, Professor, Head of the Department of Family Medicine, NKG: Ph.D. student.

## Pre-publication history

The pre-publication history for this paper can be accessed here:

http://www.biomedcentral.com/1471-2458/14/223/prepub
